# Artificial Intelligence-Based Echocardiography in Pulmonary Arterial Hypertension

**DOI:** 10.1016/j.chest.2025.06.052

**Published:** 2025-08-26

**Authors:** Bettia Celestin, Shadi P. Bagherzadeh, Everton Santana, Matthew Frost, Mathias Iversen, Frida N. Hermansson, Andrew Sweatt, Roham T. Zamanian, Yoran M. Hummel, Gabriela Gomez Rendon, Joseph Yen, Marinella Sandros, Michael Salerno, Francois Haddad

**Affiliations:** a Division of Cardiovascular Medicine, Department of Medicine, Stanford University, Palo Alto, CA; b Division of Pulmonary, Allergy and Critical Care Medicine, Department of Medicine, Stanford University, Palo Alto, CA; c Stanford Cardiovascular Institute, Palo Alto, CA; d Us2.ai, Singapore, Singapore; e Johnson & Johnson, Titusville, NJ

**Keywords:** deep learning, echocardiography, pulmonary hypertension, right heart

## Abstract

**Background:**

Echocardiography is central when assessing pulmonary hypertension (PH), but manual interpretation can be time-consuming and prone to error.

**Research Question:**

Is a fully automated deep learning (DL) workflow in echocardiography reliable when assessing PH?

**Study Design and Methods:**

This study had 2 parts: the first determined the bias and precision of DL reads by using Us2.ai software version 1.4.5 with core laboratory readers as the reference; the second part assessed the ability of DL to discriminate milder PH in patients referred for right heart catheterization (mean pulmonary artery pressure between 20 and 35 mm Hg). The first cohort (case-control) included 213 healthy individuals and 221 patients with pulmonary arterial hypertension. Parameters included peak tricuspid regurgitation velocity (TRV), right ventricular basal diameter, tricuspid annular plane systolic excursion, right atrial area, and right ventricular fractional area change (RVFAC). The referral cohort included 196 patients, with 171 patients having measurable peak TRV signals. Robust measures of bias and precision were reported, and area under the curve (AUC) analysis assessed discrimination.

**Results:**

In patients with pulmonary arterial hypertension, mean age was 48 years, 78% were female, and mean pulmonary artery pressure was 52 mm Hg. No significant bias was observed for peak TRV (0.90%; 95% CI, –0.17 to 1.57), right atrial area (1.71%; 95% CI, 0.59 to 3.34), and tricuspid annular plane systolic excursion (1.28%; 95% CI, –0.51 to 3.18), while RVFAC exhibited a significant bias of 11.46% (95% CI, 8.43 to 14.74). For all measurements except RVFAC, robust percentile precision remained below 15%. In the case-control cohort, peak TRV had AUCs of 0.99 and 0.98 for core laboratory and DL reads, respectively. The AUC for PH detection in the referral cohort was 0.79 for clinical laboratory reads and 0.75 for DL reads (*P* = .068).

**Interpretation:**

A fully automated DL workflow for echocardiography in PH is promising and likely to improve efficiency in clinical practice.

## Graphical Abstract

**Figure:**
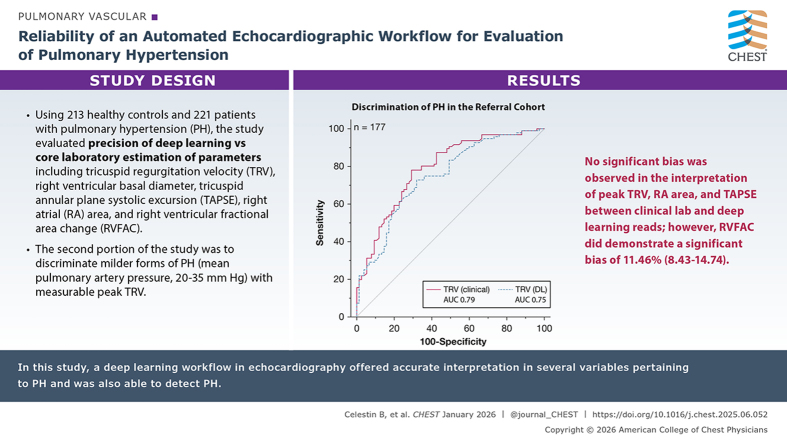

FOR EDITORIAL COMMENT, SEE PAGE 16

**Take-Home Points**

**Research Question:** Is a fully automated deep learning workflow in echocardiography reliable for assessing echocardiograms in pulmonary hypertension?

**Results:** The deep learning method exhibited low bias and good precision for peak tricuspid regurgitation velocity (TRV), right ventricular basal diameter, tricuspid annular plane systolic excursion, and TRV; in addition, its clinical value for detecting pulmonary hypertension, primarily relying on peak TRV, was comparable to core laboratory and clinical reads.

**Interpretation:** A fully automated workflow for right heart analysis was feasible and provided clinically reliable measures for peak TRV, right ventricular basal diameter, tricuspid annular plane systolic excursion, and TRV.

Echocardiography plays a key role in the assessment of patients with pulmonary hypertension (PH).^1^, ^2^, ^3^, ^4^, ^5^, ^6^, ^7^ The 2022 European Society of Cardiology/European Respiratory Society (ESC/ERS) guidelines for PH included several echocardiographic metrics in diagnostic and risk stratification algorithms, such as peak tricuspid regurgitation velocity (TRV), tricuspid annular plane systolic excursion (TAPSE), right ventricular (RV) basal diameter, and right atrial (RA) area.^1^

Echocardiographic assessment of the right heart can be time-consuming and is prone to human error, largely due to its asymmetric shape, prominent trabeculations, and abnormal septal motion.^8^, ^9^, ^10^, ^11^, ^12^ To address these challenges, deep learning (DL) has been proposed to improve the reproducibility of echocardiographic measurement.^13^, ^14^, ^15^ For the left heart, DL has proven useful for quantifying ejection fraction, longitudinal strain, and ventricular or atrial volumes.^8^^,^^16^, ^17^, ^18^ In contrast, only a limited number of studies have examined the right heart.^15^^,^^19^, ^20^, ^21^ One such study, by Hsia et al,^22^ used artificial intelligence-derived measurements of RV fractional area change (RVFAC) and RV free-wall strain to predict RV systolic dysfunction via cardiac MRI.

To our knowledge, no study to date has evaluated a fully automated workflow for right heart analysis in PH. The Us2.ai platform, a vendor-independent software, has shown reliable measurements of left ventricular volumes, ejection fraction, and Doppler metrics. The platform now includes analysis of many right heart parameters, including peak TRV, TAPSE, and RV/RA parameters. The objectives of the current study were twofold: (1) to determine whether DL can provide reliable measurements of peak TRV and RV/RA parameters; and (2) to compare DL and clinical discrimination of PH in a referral cohort.

## Study Design and Methods

### Study Design

**Figure 1:**
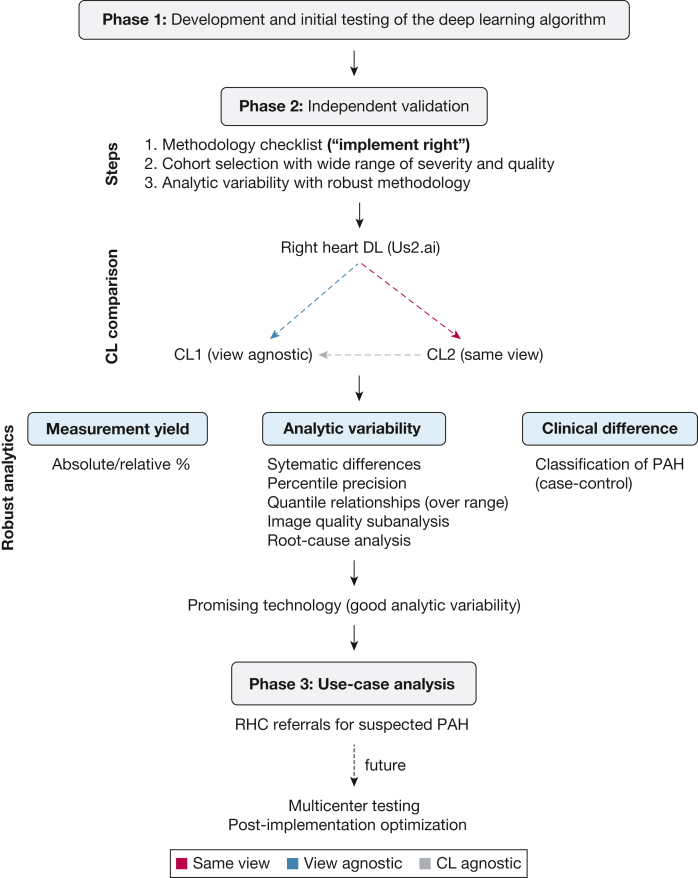
Study design and analytics. Prior to the design of the study, a robust analytic pipeline was developed for duplicate analysis. Both the DL and CL2 readers were compared with the CL1 reader (blue and gray arrows). The DL reader was also compared with the same-view CL reader (red arrow). Duplicate analysis involved the following measures: relative yield, systematic differences and scaled precision measures, quality subanalysis, quantile relationship with the range of measurements, and clinical implication. CL = core laboratory; CL1 = view-agnostic core laboratory; CL2 = same-view core laboratory; DL = deep learning; PAH = pulmonary arterial hypertension; RHC = right heart catheterization.

The study had 3 main parts (Fig 1). The first part assessed the bias and precision of DL compared with core laboratory (CL) readings in healthy volunteers and patients with pulmonary arterial hypertension (PAH); the second part evaluated DL performance in patients referred for right heart catheterization (RHC) due to suspected PAH. The third part involved a CL reader reanalyzing 50 randomly selected studies. During project planning, we created a checklist (e-Table 1) to define use-case questions, minimize selection bias, and outline a statistical analysis plan.

The study was approved by Stanford’s institutional review board (protocol number 69090). The project was approved by Stanford University institutional review board (protocol 69090, Retrospective Longitudinal Analysis of Automated Read of Transthoracic Echocardiograms in Pulmonary Hypertension).

### Study Population

#### Part 1: Case-Control Cohort

The first part of the study included healthy individuals and patients with PAH. Apparently healthy individuals were prospectively recruited in the San Francisco Bay Area as part of the Stanford Aging Study, an ongoing study started in 2009. Consecutive volunteers completed standardized questionnaires covering acute illness, exercise limitations, chest pain, and recent hospitalizations. During the early study period (2009-2011), 255 individuals responded to the initial flyer advertisement. Of these, 36 were excluded based on questionnaire responses for the following reasons: established atherosclerotic disease (n = 15), stage C heart failure (n = 4), history of atrial fibrillation (n = 2), diabetes mellitus (n = 8), current smoking (n = 3), and severe obesity (BMI > 35 kg/m^2^; n = 4). Among the 219 participants who met the screening criteria, 4 with stage B heart failure (per American Society of Echocardiography [ASE] guidelines) and 2 with ascites and nodules (later diagnosed as malignancies) were further excluded.

**Figure 2:**
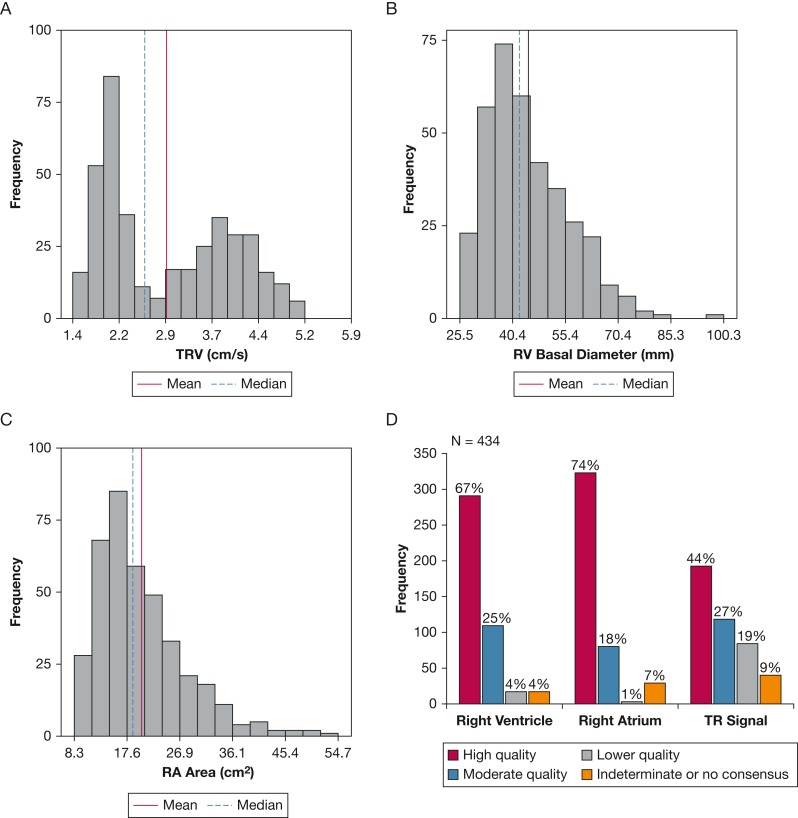
A-D, Distribution of echocardiographic characteristics and image quality. There was a wide distribution of echocardiographic parameters in the study population as shown for peak TRV (A), RV basal diameter (B), and RA area (C). D, The quality distribution for Doppler and 2D images is also shown. RA = right atrial; RV = right ventricular; TR = tricuspid regurgitation; TRV = tricuspid regurgitation velocity.

A total of 221 patients with PAH, confirmed according to ESC/ERS guidelines,^1^ were selected from the Stanford PH registry. All patients underwent RHC within 2 weeks of echocardiography, with 177 (80%) undergoing the procedure within 1 week. This cohort exhibited a broad range of chamber enlargement and ventricular dysfunction (Fig 2).

#### Part 2: RHC Referral Cohort

The referral cohort was selected from the Stanford CardioShare Registry, which compiles data on patients who underwent RHC and echocardiography from January 2005 onward. From this registry, we identified 351 patients referred to the PH center for suspected PAH who had undergone echocardiography within 3 months prior to RHC. Fifty-six patients referred specifically for left heart or chronic lung disease were excluded. In addition, to focus on mild PH, 99 patients with mean pulmonary artery pressure (MPAP) > 35 mm Hg were excluded. The final cohort comprised 196 patients.

### DL (Us2.ai) Reads

Us2.ai software version 1.4.5 was used for the analysis. The software was developed by using Digital Imaging and Communications in Medicine (ie, DICOM) files. The RV segmentation algorithms were trained on 5,498 images from 1,740 patients; TAPSE on 2,171 images from 1,512 patients; continuous wave tricuspid regurgitation (TR) method on 6,336 images from 6,336 patients; and RA segmentation on 3,528 images from 1,912 patients.

The DL workflow was previously described by Tromp et al.^8^^,^^18^ In brief, steps for the development of the DL models included: (1) view classification and annotation of the DICOM files by expert readers; (2) segmentation models based on a U-Net-style architecture with a sigmoid output layer, trained with the combined binary cross-entropy and Dice loss function; and (3) development of confidence scores based on view quality and measurement quality, with only values meeting both criteria being reported. After uploading the de-identified studies, an automated workflow performed view classification, view selection, segmentation, and measurements of peak TRV, RV basal diameter, RV end-diastolic area (RVEDA), RV end-systolic area (RVESA), RVFAC, and RA area.

### CL Reads

CL analyses were conducted by 2 Level 3 readers (B. C. and F. H.). One author (B. C.) served as the view-agnostic reader (CL1 reads), while the second author (F. H.) evaluated the same view selected by Us2.ai (CL2 reads) (Fig 1). Studies were analyzed following the recommendations of the ASE and acquired on Philips ultrasound systems.^23^^,^^24^ The right ventricle was measured by using RV-focused views, with the RV basal diameter assessed parallel to the annulus. Peak TRV was measured at the modal frequency by the CL readers, and no agitated saline was used. Doppler and 2-dimensional images were graded on a Likert quality scale (e-Table 2): 1 (non-interpretable), 2 (poor), 3 (suboptimal but interpretable), 4 (good), and 5 (excellent). For analysis, quality was categorized as good (scores 4-5), moderate (score 3), or low (scores 1-2). Examples of TR signal and RV view image qualities are presented in e-Figure 1.

At the end of the study, the view-agnostic reader (B. C.) randomly selected and analyzed 50 studies; this reader was masked to the initial results but aware of the results of the case-control analysis.

### Statistical Analysis

Analysis was conducted by using Python 3.11.5 (Python Software Foundation) and RStudio version 4.1.2 (Posit PBC). Numerical values are presented as mean ± SD or median with interquartile range for skewed data. Categorical variables are expressed as frequency counts (n/N) and percentages.

The relative yield of DL was calculated as the proportion of CL1 reads. CL1 served as the primary reference for comparing DL and CL2, and a secondary analysis was conducted to compare DL and CL2 directly. Associations between measurements were assessed by using the Spearman correlation, followed by duplicate analysis of measurement differences on a nominal or relative scale.

Various methods exist to report inter-reader variability (e-Table 3). For the current study, we focused on robust measures of bias and precision, as they are less sensitive to outliers and provide more realistic estimates of reference change values. Bias was reported as the median difference, whereas precision was calculated as one-half the difference between the 84th and 16th percentiles (½ × [P84 – P16]) and scaled by dividing by √2 to account for individual measurement variability.^25^ A bootstrap method with 1,000 resamples was used for 95% CIs. To analyze differences across the measurement range, quantile regression was performed at the 0.5 (median), 0.16, and 0.84 quantiles. The impact of image quality was assessed by using the Kruskal-Wallis test for median differences and the Levene test for equality of variance.

PH discrimination was evaluated by using the area under the curve (AUC), with differences assessed by using the method of DeLong et al.^26^ In the first cohort, patients with PAH were compared with age- and sex-matched healthy volunteers, and the referral cohort classified PH based on an MPAP > 20 mm Hg.

## Results

### Part 1: Case-Control Cohort

The first cohort included 213 healthy adults and 221 patients with PAH (Table 1). In the healthy group, mean ± SD age was 55 ± 17 years, 54% self-identified as male, and 85% self-identified as White. In the PAH group, mean age was 48 ± 14 years, 78% self-identified as female, and 56% self-identified as White.

**Table 1:** Study Population Characteristics

Characteristic	Healthy Group (n = 213)	PAH Group (n = 221)
Age, y	55 ± 17	48 ± 14
Female sex	99 (46)	173 (78)
Race		
White	178^a^ (85)	123 (56)
Asian	24^a^ (11)	21 (10)
Black	4^a^ (2)	8 (4)
Other (not specified)	4^a^ (2)	69 (31)
BMI, kg/m^2^	24.5 ± 3.4	29.0 ± 6.9
Systolic BP, mm Hg	119 ± 14	115 ± 18
Diastolic BP, mm Hg	74 ± 10	72 ± 13
Heart rate, beats/min	61 ± 10	80 ± 15
Echocardiographic measurements		
Peak TRV, m/s	2.1 ± 0.5	4.1 ± 0.7
RV basal diameter, mm	37.8 ± 8.3	54.8 ± 10.2
TAPSE, mm	25.1 ± 3.6	16.1 ± 4.8
RA area, cm^2^	15.9 ± 4.1	23.3 ± 9.3
RVEDA, cm^2^	22.2 ± 5.5	37.4 ± 11.6
RVESA, cm^2^	13.3 ± 3.4	30.5 ± 11.2
RVFAC, %	39.9 ± 3.6	19.7 ± 6.5
LVEF, %	62.0 ± 4.8	64.7 ± 7.8

Data are presented as mean ± SD or No. (%). LVEF = left ventricular ejection fraction; PAH = pulmonary arterial hypertension; RA = right atrial; RV = right ventricular; RVEDA = right ventricular end-diastolic area; RVESA = right ventricular end-systolic area; RVFAC = right ventricular fractional area change; TAPSE = tricuspid annular plane systolic excursion; TRV = tricuspid regurgitation velocity.

a n = 210.

The most common cause of PAH was idiopathic (48%). The MPAP was 52 ± 14 mm Hg, mean pulmonary vascular resistance was 12.5 ± 6.2 Wood units, and the mean Registry to Evaluate Early and Long-Term PAH Disease Management (REVEAL) Lite 2 risk score was 7.9 ± 3.1 (e-Table 4A). The medication profiles are presented in e-Table 4B.

### Echocardiography

There was a wide range of peak TRV and RV measurements in the cohort (Figs 2A-2C). Peak TRV and TAPSE achieved the highest yield (> 90%), followed by RA area, RV basal diameter, RVEDA, and RVESA with yields between 80.8% and 87.1%, and RVFAC with a yield of 78.5% (Table 2). High-quality images were most common for RA images (74%), followed by RV images (67%) and peak TR signals (44%) (e-Table 5, Fig 2D).

**Table 2:** Right Heart and Tricuspid Velocity Parameter Yield (n = 434)

Characteristic	CL Reads	DL Reads	Common Reads	Relative Yield (%)
Peak TRV, m/s	396	403	370	93.4
RV basal diameter, mm	422	357	341	80.8
TAPSE, mm	316	319	302	95.6
RA area, cm^2^	426	376	371	87.1
RVEDA, cm^2^	408	351	331	81.1
RVESA, cm^2^	408	351	332	81.3
RVFAC, %	413	329	324	78.5

TAPSE was not acquired for the entire cohort due to limited protocol in some patients. CL = core laboratory; DL = deep learning; RA = right atrial; RV = right ventricular; RVEDA = right ventricular end-diastolic area; RVESA = right ventricular end-systolic area; RVFAC = right ventricular fractional area change; TAPSE = tricuspid annular plane systolic excursion; TRV = tricuspid regurgitation velocity.

### Associations Between DL and CL Reads

Associations between DL and CL reads were assessed by using the Spearman correlation (e-Table 6). Very strong associations were noted for peak TRV (*Ρ* = .90), RVESA (*Ρ* = .89), and RA area (*Ρ* = .86), while strong associations were noted for RV basal diameter (*Ρ* = .76), TAPSE (*Ρ* = .78), and RVFAC (*Ρ* = .77). In general, higher correlations were observed between DL and CL2 (same view) and among CL readers (CL1 and CL2). In the PAH group, associations remained strong and were consistent across comorbidity subgroups.

### Systematic Differences (“Bias”) and Percentile Precision

**Figure 3:**
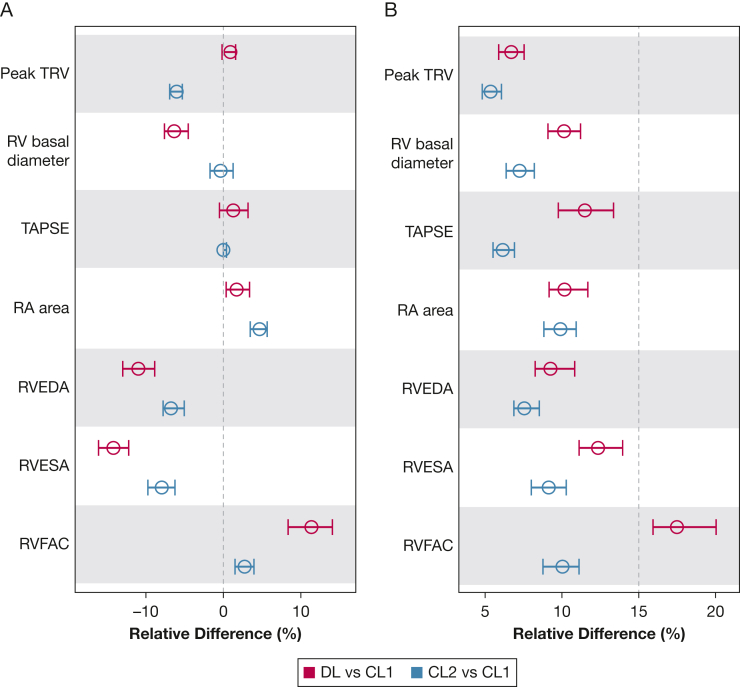
Bias and precision measures compared with the CL1 reader. Systematic differences (A) and scaled percentile precision (B) for peak TRV, RV basal diameter, TAPSE, RA area, RVEDA, RVESA, and RVFAC within the automated DL and manual CL2 reads compared with the CL1 reads. CL1 = view-agnostic core laboratory; CL2 = same-view core laboratory; DL = deep learning; RA = right atrial; RV = right ventricular; RVEDA = right ventricular end-diastolic area; RVESA = right ventricular end-systolic area; RVFAC = right ventricular fractional area change; TAPSE = tricuspid annular plane systolic excursion; TRV = tricuspid regurgitation velocity.

The relative bias remained below 5% for peak TRV, TAPSE, and RA area in both DL and CL2 reads (Fig 3A, Table 3). For RV basal diameter, DL reads slightly underestimated diameters (–6.35%; 95% CI, –7.60 to –4.53), while both CL2 and DL reads recorded smaller RV areas and higher RVFAC. With the exception of RVFAC in DL reads, precision remained below 15% (Fig 3B, Table 3). Table 3 provides detailed nominal and relative values, and e-Table 7 presents DL and CL2 comparisons.

**Table 3:** Systematic Differences and Scaled Percentile Precision for the DL and CL2 Reads vs the CL1 Reads

Measure	Comparator to CL1	Systematic Difference	Percentile Precision
Nominal differences			
Peak TRV	DL	0.02 (–0.004 to 0.04)	0.18 (0.16 to 0.20)
	CL2	–0.15 (–0.18 to –0.14)	0.16 (0.14 to 0.18)
RV basal diameter	DL	–2.58 (–3.34 to –1.79)	4.63 (3.91 to 5.18)
	CL2	–0.36 (–1.72 to 1.25)	3.25 (2.85 to 3.66)
TAPSE	DL	0.22 (–0.13 to 0.65)	2.21 (1.96 to 2.53)
	CL2	0 (0 to 0.40)	1.27 (1.12 to 1.41)
RA area	DL	0.33 (0.09 to 0.58)	1.90 (1.66 to 2.18)
	CL2	0.76 (0.60 to 1.20)	1.89 (1.68 to 2.16)
RVEDA	DL	–2.90 (–3.35 to –2.23)	2.64 (2.30 to 3.04)
	CL2	–1.80 (–2.05 to –1.40)	2.19 (1.98 to 2.46)
RVESA	DL	–2.50 (–2.78 to –2.05)	2.24 (2.02 to 2.42)
	CL2	–1.45 (–1.71 to –1.20)	1.66 (1.41 to 1.88)
RVFAC	DL	3.69 (2.61 to 4.48)	5.44 (4.79 to 6.10)
	CL2	0.70 (0.39 to 1.00)	2.73 (2.46 to 2.98)
Relative difference			
Peak TRV	DL	0.90 (–0.17 to 1.57)	6.70 (5.88 to 7.53)
	CL2	–6.02 (–6.93 to –5.30)	5.35 (4.81 to 6.06)
RV basal diameter	DL	–6.35 (–7.60 to –4.53)	10.13 (9.08 to 11.21)
	CL2	–0.36 (–1.72 to 1.25)	7.24 (6.37 to 8.20)
TAPSE	DL	1.28 (–0.51 to 3.18)	11.49 (9.76 to 13.36)
	CL2	0 (0 to 1.57)	6.15 (5.52 to 6.90)
RA area	DL	1.71 (0.59 to 3.34)	10.17 (9.11 to 11.69)
	CL2	4.67 (3.35 to 5.67)	9.85 (8.77 to 10.83)
RVEDA	DL	–11.0 (–12.99 to –8.41)	9.24 (8.32 to 10.68)
	CL2	–6.89 (–7.82 to –5.27)	7.46 (6.81 to 8.47)
RVESA	DL	–14.29 (–16.15 to –12.51)	12.35 (10.96 to 13.97)
	CL2	–8.18 (–9.79 to –6.25)	9.14 (7.89 to 10.22)
RVFAC	DL	11.46 (8.43 to 14.74)	17.34 (15.65 to 19.56)
	CL2	2.70 (1.52 to 4.01)	10.01 (8.78 to 11.03)

Data are presented as median and 95% CI. CL1 = view-agnostic core laboratory; CL2 = same-view core laboratory; DL = deep learning; RA = right atrial; RV = right ventricular; RVEDA = right ventricular end-diastolic area; RVESA = right ventricular end-systolic area; RVFAC = right ventricular fractional area change; TAPSE = tricuspid annular plane systolic excursion; TRV = tricuspid regurgitation velocity.

Reference limits from the healthy cohort provide additional context for interpreting analytic differences. The median values and 5th to 95th CIs for this group are presented in e-Table 8. For peak TRV, RV basal diameter, RA area, and TAPSE, reader differences were minimal and closely aligned with recent World Alliance Societies of Echocardiography or ASE reference values.^2^^,^^3^ However, RV areas were larger, with even greater differences in CL1 reads. In addition, the higher imprecision of RVFAC in DL reads resulted in wider confidence limits.

### Factors Influencing Relative Difference Between Measures

**Figure 4:**
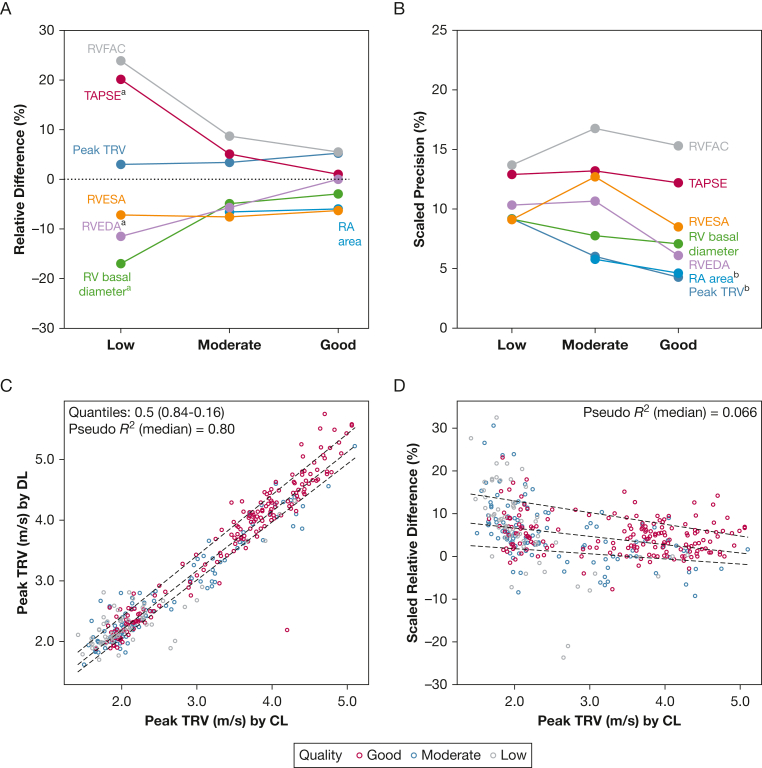
A-D, Quality and analytic variability. A, The influence of image quality on the relative difference (bias) in measures. ^a^Significant differences in quality were noted for RV basal diameter, RVEDA, and TAPSE. B, The influence of image quality on scaled precision. ^b^Significant differences were noted for peak TRV and RA area. C, The association between CL- and DL-measured peak TRV. D, The quantile regression for scaled relative differences in peak TRV measures. An extreme point has been removed from the relative difference to allow better visualization. CL = core laboratory; DL = deep learning; RA = right atrial; RV = right ventricular; RVEDA = right ventricular end-diastolic area; RVESA = right ventricular end-systolic area; RVFAC = right ventricular fractional area change; TAPSE = tricuspid annular plane systolic excursion; TRV = tricuspid regurgitation velocity.

Relative differences varied by image quality and measurement range (Figs 4A, 4B). Lower quality signals resulted in significantly greater bias for RV basal diameter, RVEDA, and TAPSE, as well as lower precision for RA area and peak TRV. There was a strong relationship between the DL and CL reads for Peak TRV (Fig 4C). Relative precision did, however, vary across the measurement range (Fig 4D). This variation can be modeled by using precision-range equations (1/2× [84th – 16th quantiles]), with examples provided at lower and higher representative values (e-Table 9). In some cases, overall group precision exceeded equation-derived precision, particularly when variance was unequal (eg, RV basal diameter and RA area).

To better understand the factors associated with large differences between the DL and CL2 reads, cases outside the 95th CI were analyzed. Peak TRV estimation at non-modal frequencies accounted for most discrepancies, with low-quality signal estimations explaining the remainder. In only 2 instances was the TR signal misclassified as a mitral regurgitation signal. For RV area measurements, segmentation was suboptimal in severely dilated and spherical right ventricles with a dominant RV apex. In addition, difficulty in classifying the systolic phase in the presence of abnormal septal motion was associated with underestimation of RVESA. For the RA area, variability was associated with a prominent septal bulge, significant pericardial effusion, translational motion, or, in rare cases, incorrect view selection (one instance).

### Classification of PAH and Relationship With Hemodynamics

Peak TRV measured by DL and CL1 reads showed excellent discrimination of PAH status, with AUCs of 0.98 and 0.99, respectively (*P* < .001). Although RA and RV areas had similar discrimination for PAH, CL reads of RV basal diameter, TAPSE, and RVFAC achieved higher AUCs (e-Table 10). Because patients with PAH in this cohort were younger and predominately female, the healthy volunteers were age- and sex-matched with patients with PAH prior to receiver-operating characteristic (ROC) curve analysis. In further support of the classification for PAH, hemodynamic associations were also analyzed. In the PAH cohort, pulmonary systolic pressure was moderately associated with DL- or CL-derived RV systolic pressure (Spearman *r* = 0.54 and 0.47, respectively, *P* < .001; *P* = .35 for difference) (e-Fig 2).

### Part 2: Referral Cohort

The median age in the referral cohort was 62 ± 16 years, and 47% were male (e-Table 11). The median time from echocardiography to RHC was 23 days (interquartile range, 4-48 days). The median MPAP in the total cohort was 21 mm Hg (7.1); 89 patients had MPAP < 20 mm Hg, and 107 patients had 20 mm Hg < MPAP ≤ 35 mm Hg.

**Figure 5:**
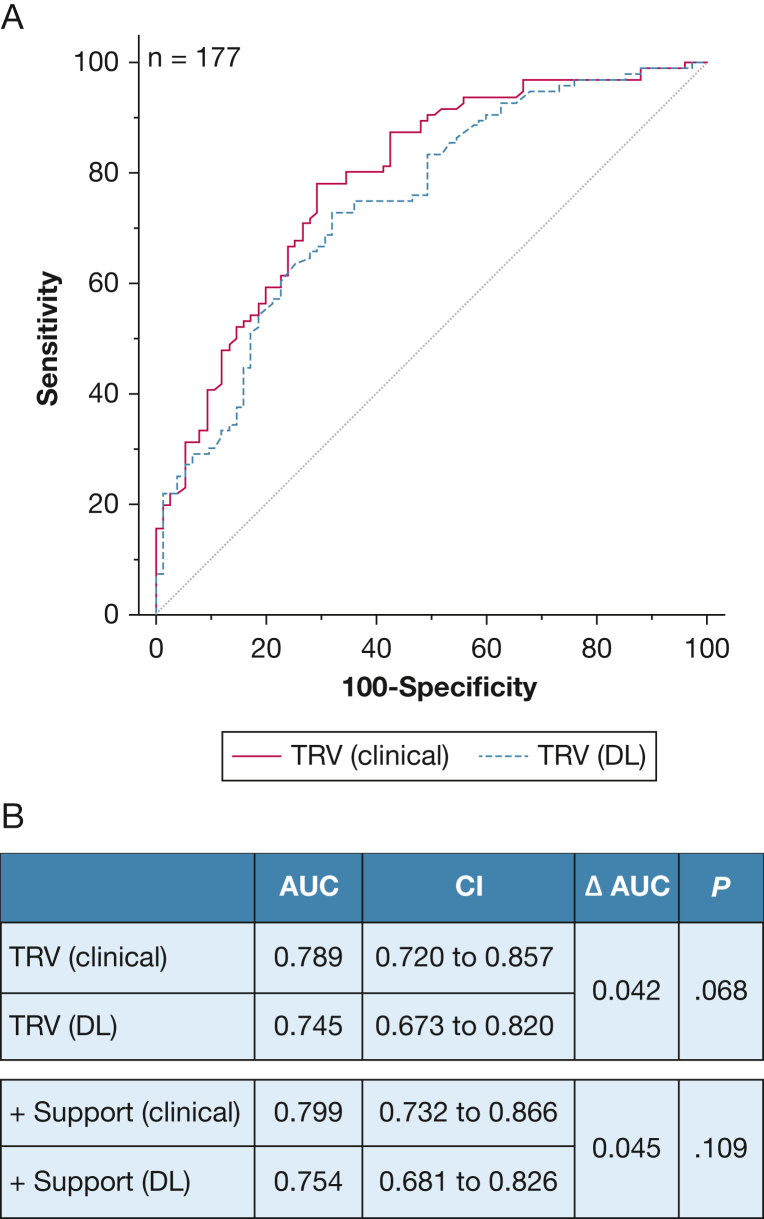
A, B, Discrimination of PH in the cohort referred to RHC for suspected PH. A, AUC of peak TRV for the diagnosis of PH based on an MPAP > 20 mm Hg for DL and clinical reads. B, Comparison of AUC of clinical reads and DL for peak TRV with and without supporting criteria. AUC = area under the curve; DL = deep learning; MPAP = mean pulmonary artery pressure; PH = pulmonary hypertension; RHC = right heart catheterization; TRV = tricuspid regurgitation velocity.

Of the 196 patients, 171 had peak TRV available by both methods. There was no statistically significant difference between the ROC curve of peak TRV from CL reports (AUC, 0.79; 95% CI, 0.72-0.85) and the ROC curve of DL reads (AUC, 0.75; 95% CI, 0.67-0.81; *P*_difference_ = .068). For DL reads, peak TRV was the most discriminative metric (AUC, 0.74; 95% CI, 0.67-0.81), followed by RV basal diameter (AUC, 0.64; 95% CI, 0.56-0.72), RA area (AUC, 0.62; 95% CI, 0.54-0.69), and RVFAC (AUC, 0.59; 95% CI, 0.51-0.67). Supporting criteria as suggested by the ESC/ERS guidelines (e-Table 12) did not improve model fit of clinical reads and modestly improved DL discrimination (*P* = .043). Although the AUC was nominally higher for clinically informed reads, the *P* value was .109 (Fig 5).

### Repeated Measures of CL1 Reader

In a masked reanalysis by CL1 (n = 50), the second reads generally showed lower peak TRV, smaller RVESA, and higher RVFAC. For peak TRV, relative bias was 4.5% with its bootstrapped 95% CI (–6.9% to 2.3%) with percentile precision of 5.8% (4.0% to 7.4%); for RVESA, relative bias was –8.0% (–11.4% to –6.9%) with percentile precision of 5.8% (3.9% to 8.2%); and for RVFAC, relative bias was 14.1% (9.5% to 22%) with high percentile precision (21.2%; 16.6% to 25.3%).

## Discussion

The current study showed that a fully automated DL workflow in echocardiography provides good accuracy (low bias) and acceptable precision (coefficient of variation within 15%) for measurements, including peak TRV, RV basal diameter, TAPSE, and RA area. The discrimination of PAH using DL with peak TRV also had good performance in both the case-control and the referral cohorts. The study also provides extensive data on inter-reader variability, helping define thresholds for meaningful serial changes in echocardiography.

DL approaches for the right heart are being actively investigated to improve efficiency and reduce inter-reader variability. Both segmentation-based and nonsegmentation-based methods have been studied. Segmentation-based approaches first determine cardiac contours prior to performing geometric analysis, whereas nonsegmentation methods analyze pixel-based function without segmenting the chambers. Several commercial software options support semi-automated analyses, including QLab, TomTec (Philips Healthcare), Velocity Vector Imaging (Siemens Healthineers), LVivo RV (DiA Imaging Analysis), and EchoInsight (Epsilon Imaging). Studies have highlighted the potential of these methods. Hsia et al^22^ showed that 2-dimensional quantification of RVFAC, RV free-wall strain, and TAPSE using LVivo RV software could predict low RV ejection fraction (< 40%) based on cardiac MRI. Liu et al^19^ showed the feasibility of artificial intelligence-based RV assessment using transesophageal echocardiography in a perioperative setting with EchoInsight software. Better reproducibility of automated tracing compared with clinical reads has also been shown in patients with congenital heart disease.^20^^,^^27^ In addition, nonsegmentation-based methods have been promising. Shad et al^14^ predicted right heart failure following left ventricular assist device implantation using temporally resolved data, and Tokodi et al^15^ predicted 3-dimensional RV ejection fraction from 2-dimensional 4-chamber images. Emerging 3-dimensional methods for RV analysis have shown strong correlations with volumes measured by cardiac MRI.^21^^,^^28^

For automated methods to be clinically valuable, they must display minimal systematic differences (bias) and acceptable precision, typically < 15%.^29^^,^^30^ These thresholds were met for the 4 measures recommended in the ESC/ERS guidelines: peak TRV, RV basal diameter, TAPSE, and RA area. However, greater variability was observed for RVFAC, which also exhibited a lower measurement yield.

Various methods exist to analyze systematic differences (“bias”) and random variation (“precision”) in repeated measurements. In imaging, the Bland-Altman method is commonly used to determine limits of agreement, defined as the mean bias ± 1.96 SD. The current study used median bias and percentile precision, which defines precision as one-half the difference between the 84th and 16th percentiles, corresponding to –1 and 1 SD in a normal distribution. Compared with SD, this approach reduces the influence of outliers. The root mean square method, commonly used in laboratory medicine, provides a global measure of both precision and bias. In the presence of systematic bias, the root mean square is always larger than the Bland-Altman relative SD or percentile precision.

Distinguishing bias from precision has important clinical implications. Systematic differences can often be addressed through calibration. In the current study, these differences were most pronounced in RV basal diameter and RV area measurements, primarily due to challenges in defining myocardial border or cardiac phase. For peak TRV, variation between CL readers depended on whether peak velocity was estimated at the modal frequency. Differences in RA area were mainly related to extension of the traces in the RA septum. Systematic differences were observed not only between DL and CL1 reads but also between the 2 CL reads. This led to a re-read of 50 studies, which revealed smaller ventricular dimensions and lower peak TRV on the second reads, highlighting the importance of calibration. It also emphasized the greater imprecision of RVFAC compared with RV area measures as it combines the uncertainty of 2 measures.

When using the same calibrated method, precision is essential for longitudinal monitoring. Both precision and within-subject biological variation set the thresholds for meaningful change, known as reference change values (RCVs). RCVs are calculated as RCV = √2 × Z score × CV_total_, where *CV*_*total*_ is the combined analytic and biological variation. For example, with a precision of 10% and a *Z* score of 1.96 (α = .05), the RCV is approximately 30% to 35%, assuming minimal bias and low biological variation. In addition, as the current study highlights, precision can vary across the measurement range, adding nuance to interpretation. Image quality and optimal acquisition affect both systematic differences and precision, highlighting the need for algorithms that integrate quality control and confidence scoring for measurements.

The clinical value of DL was also shown in this study for both the case-control and referral-based cohorts. Establishing the usefulness of DL in mild cases of PH is crucial, as this is where the diagnostic challenge lies. Both the DL and CL reads showed overall comparable performance. However, clinicians are aware of the indication for the study, which can introduce information bias.

The current study has several limitations. It was single center with CL studies interpreted by experienced clinicians. The analysis used just 1 vender (Philips), and thus comparison with other systems is warranted. The findings are specific to Us2.ai version 1.4.5, and future versions of this software will need validation. Although we explored detecting mild PH, future multicenter studies are needed to validate diagnostic and monitoring approaches, including longitudinal follow-up and hemodynamic response to therapy. Finally, as shown here, the feasibility and reliability of a fully automated DL workflow are influenced by image quality. Therefore, future integration of confidence levels in automated reads may help guide interpretation and clinical decision-making.

## Interpretation

This study shows the value and feasibility of a fully automated DL workflow for right heart and peak TRV analysis. Reliable measures included peak TRV, RV basal diameter, TAPSE, and RA area. The study quantified analytical variability, aiding in the interpretation of reference change values. Future studies will test the workflow’s value for detecting and risk stratifying PH, including integration with novel machine learning approaches.^31^

## Funding/Support

The study was funded by Actelion Pharmaceuticals US, Inc., a Janssen Pharmaceutical Company of Johnson & Johnson. Data acquisition and analysis were performed independently by the researchers, with scientific input provided through the collaboration.

## Financial/Nonfinancial Disclosures

The authors have reported to *CHEST* the following: F. H. received research funding from 10.13039/100004331Johnson & Johnson for investigator-initiated studies on computational methods in pulmonary hypertension. M. F., M. I., and Y. M. H. are employees of Us2.ai. G. G. R., J. Y., and M. Sandros are employees of Johnson & Johnson. None declared (B. C., S. P. B., E. S., F. N. H., A. S., R. T. Z., M. Salerno).

## Supplementary Data

e-Online Data

Audio
