# Supplementary material for: Artificial Intelligence-Based Echocardiography in Pulmonary Arterial Hypertension
Source: Chest. 2025 Aug 26;169(1):207–19. doi: 10.1016/j.chest.2025.06.052 (PMC13169424; doi:10.1016/j.chest.2025.06.052)
Supplement: e-Online Data [file mmc1.docx]

**Supplemental Material**

**Artificial Intelligence-Based Echocardiography in Pulmonary Arterial Hypertension**

*Bettia Celestin, MD, MSc; Shadi P. Bagherzadeh, MD; Everton Santana, MSc; Matthew Frost, PhD; Mathias Iversen, MSc; Frida N. Hermansson, MSc; Andrew Sweatt, MD; Roham T. Zamanian, MD; Yoran M. Hummel, PhD; G. Gomez Rendon, MD; Joseph Yen, PhD; Marinella Sandros, PhD; Michael Salerno, MD, PhD; and Francois Haddad, MD*

| **e-Table 1** IMPLEMENT-RIGHT Checklist Prior to Conducting an Independent Assessment or Validation of DL Technology | Page 3 |
| --- | --- |
| **e-Table 2** Likert Scale for Quality Grading System | Page 5 |
| **e-FIGURE 1** Examples of Grading of Image Quality | Page 7 |
| **e-Table 3** Statistical Methods Summary for Duplicate Analysis | Page 8 |
| **e-Table 4** Characteristics of the PAH Group; and Detailed Medication Profile | Page 9 |
| **e-table 5** Granular Quality Assessment of Peak TRV and Right Heart Parameters | Page 11 |
| **e-TABLE 6** Spearman Correlation of Deep Learning and Core Laboratory Reads | Page 12 |
| **e-Table 7** Systematic Differences and Scaled Percentile Precision of the DL Versus CL2 Reader | Page 14 |
| **e-TABLE 8.** Median and Percentile Range for Core Laboratory and Deep Learning Reads Compared to the World Alliance Societies of Echocardiography and American Society of Echocardiography Reference Limits and Thresholds | Page 15 |
| **e-TABLE 9** Relative Scaled Precision Across the Range of Measures (DL vs. CL1 Reader) | Page 16 |
| **e-Table 10** AUC for the Discrimination of PAH Status with Fully Automated Echocardiography | Page 17 |
| **e-FIGURE 2** Estimated Right Heart Catheterization Versus Invasively Measured Pulmonary Systolic Pressure in DL Versus Controls | Page 18 |
| **e-Table 11** Clinical and Hemodynamic Characteristics of the Use-Case Referral Cohort | Page 19 |
| **e-TABLE 12** Supporting Criteria for the Diagnosis of Pulmonary Hypertension | Page 20 |

**e-Table 1** IMPLEMENT-RIGHT Checklist Prior to Conducting an Independent Assessment or Validation of DL Technology

| Checklist |
| --- |
| 1. Comparison question: Define the questions and state which technology is being compared |
| 2. Stage of validation: Has the technology already undergone initial testing (derivation, validation, testing) during the development phase? |
| 3. Conflict of interest: Does the group conducting the comparison have a conflict of interest with the company or with regards to their own research portfolio? |
| 4. Study design and statistical analysis plan |
| - 4A. Sites: is it a single center or multicenter study? - 4B. Machines: Are the acquisitions on the same vendor or multivendor? - 4C. Selection of the cohort: Does the cohort selected have sufficient range for the metrics being evaluated? B. Does the cohort have a good range for quality ratings of imaging (signal to noise ratio)? C. Does the cohort allow testing for differences in outcome? - 4D. Comparator: Is the comparator well stated: clinical reads versus core laboratory reads? - 4E. De-identification of studies: Are the studies de-identified to minimize bias in the analysis by the core laboratory read or the deep learning (DL) software? - 4F. View selection and view comparison: Is the DL method compared on the same view or the core laboratory read independently choses his view. Are the criteria for view or signal selection well presented? - 4G. Signal quality rating: Is the signal quality objectively rated? Will this be included in a sensitivity analysis? - 4H. Are the analytic variability metrics to be compared well stated, i.e., yield, analytic variability, differences in clinical outcome? - 4I. Statistical analysis plan: Is there a predefined statistical analysis plan (SAP)? Does the SAP clearly state which metric is intend to compare: analytic variability, efficiency of analysis, clinical end-point differences? Is the plan for sensitivity analysis predefined? |
| 5. Results:   - Do the results reflect the predefined statistical analysis plan? - Are all the planned analyses presented? - For reporting yield, is the yield presented both as overall yield and sub-analyzed per signal quality? - For analytic variability, does the analysis clearly present bias and precision of reporting ideally using confidence interval? - For reporting efficiency, important to have a clear statement whether comparison also involves loading of software and which setting for comparison - For reporting outcome, important to ensure comparison on the sample cohort (signals assessed) reporting outcomes using confidence interval methodology |
| 6. Root cause analysis: Does the study address root cause analysis of failures of segmentation? |
| 7. Clinical implications:   - Does the study state clinical implications in terms of readiness for implementation? - Does the study outline potential next steps in the development phase? |

**e-Table 2** Likert Scale for Quality Grading System

**A. Tricuspid Regurgitation Doppler Signal**

| **Grading** | **Completeness** | **Modal Frequency** |
| --- | --- | --- |
| 5 | Complete | Well defined modal frequency |
| 4 | Complete | Modal frequency not as well defined |
| 3 | Peak not perfectly defined | - |
| 2 | Peak not visualized but transitions visualized | - |
| 1 | Neither peak nor transition visualized | - |

An additional quality metric was added when nonmodal frequency (e.g., beard instead of chin) was present.

**B. Right Ventricular 2D Imaging (Four-Chamber View)**

| **Grading** | **Complete Visualization of the Right Ventricle** | **Borders Well Defined** |
| --- | --- | --- |
| 5 | Yes | Yes |
| 4 | Yes | Up to 1/3 of wall segment not well defined |
| 3 | No | Up to 1/3 of wall segment not well defined |
| 2 | No | Up to 2/3 of wall not well visualized |
| 1 | No | More than 2/3 not visualized |

**C. Right Atrial 2D Images**

| **Grading** | **Complete Visualization of the Right Ventricle** | **Borders Well Defined** |
| --- | --- | --- |
| 5 | Yes | Yes |
| 4 | Yes | Up to 1/3 of wall segment not well defined |
| 3 | No | Up to 1/3 of wall segment not well defined |
| 2 | No | Up to 2/3 of wall not well visualized |
| 1 | No | More than 2/3 not visualized |

**D. M-Mode for TAPSE**

| **Grading** | **Clear Delineation of a Signal** | **One Clear Single Line** |
| --- | --- | --- |
| 5 | Yes | Yes |
| 4 | Yes | No |
| 3 | Yes, but less clear | No |
| 2 | Barely | No |
| 1 | No | No |

**e-Figure 1** Examples of Grading of Image Quality

**
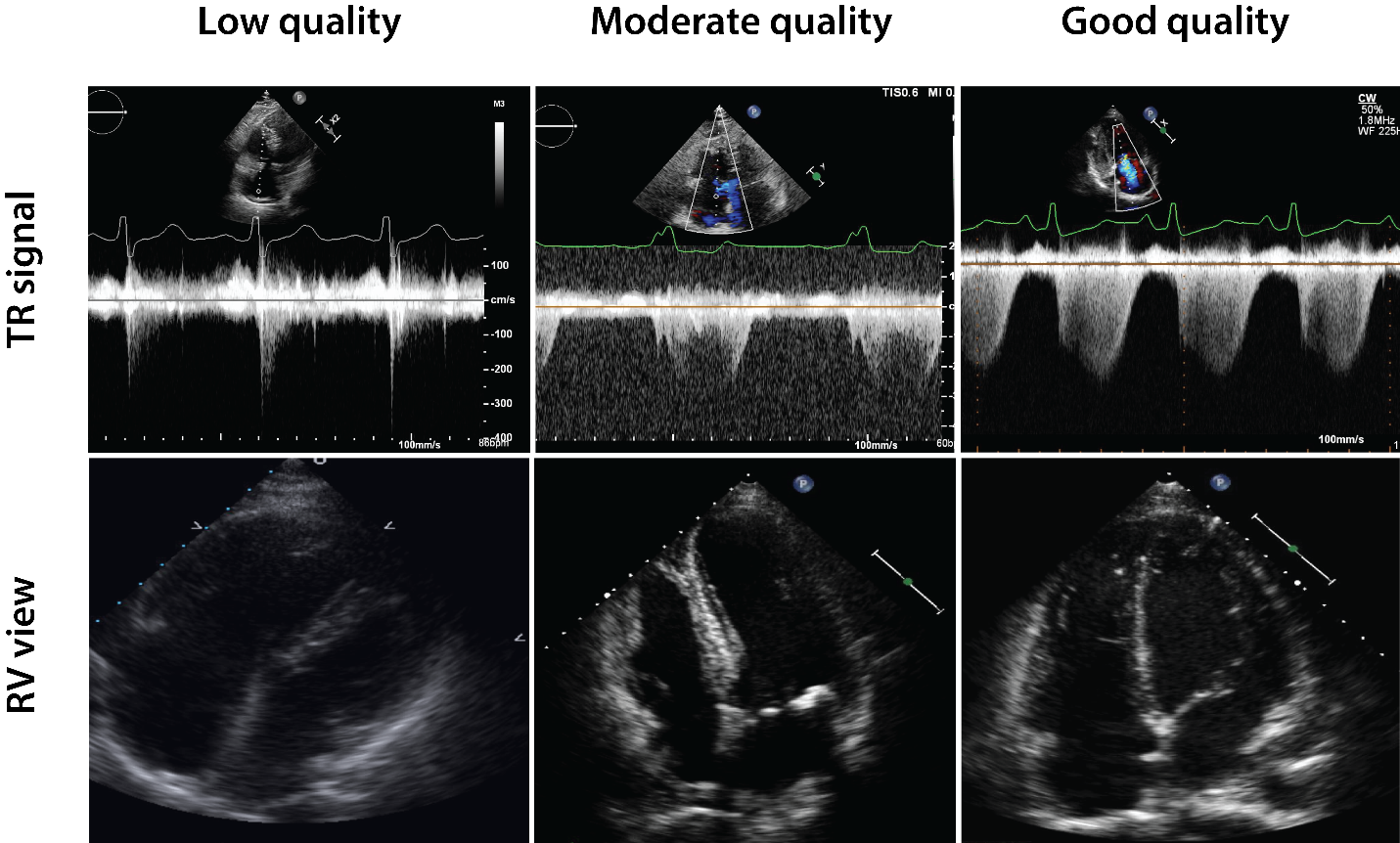
**Examples of the final classification of image quality. For the TR signal, the low-quality images show a poorly defined peak, which precludes reliable quantification; moderate-quality images can usually be assessed reliably. The low-quality RV image was the best view achieved during the study but was a modified, rather than a focused, RV view with poorly defined wall thickness borders; the moderate-quality image, although the best during the study, is sub-optimally rotated with an incompletely visualized apical border. The good-quality RV view is focused with well-defined borders. RV = right ventricular; TR = tricuspid regurgitation.

**e-Table 3** Statistical Methods Summary for Duplicate Analysis

**A. Method Yield**

Relative yield = number measured new method/number measured reference method

**B. Simple Measure of Associations**

Spearman correlation assesses the consistency of associations

**C. Differences (d) for Duplicate Measures (m_1_ and m_2_)**

| **Scale** | **Formula** | **Comment** |
| --- | --- | --- |
| Nominal | d_n_ = m_2_ - m_1_ | Sometimes referred to incorrectly as ‘absolute’ |
| Relative | d_r_ = (m_2_ - m_1_) / mean | Normalized to the mean |
| Scaled | d_scaled_ = d / √2 | √2 accounts for variability in two separate measurements |

**D. Analytic Variability Measures for Duplicate Measures**

| **Measure** | **Formula** | **Comment** |
| --- | --- | --- |
| Systematic differences ‘bias’ | mean or **median** of difference (d) | Requires a reference method  Median less sensitive to outliers |
| Bland-Altman precision | SD of d (unscaled) | Commonly used for focus on total variation |
| Scaled precision | Parametric: SD of d_scaled_  **Robust: ½ (P84–P16)** | Precision measures are usually scaled  Percentile precision uses a normal SD equivalent  Robust less sensitive to outliers |
| RMS ‘CV’ | √ ∑ (d_scaled_i_ ^2^) /n) | Captures both precision and bias (mean not subtracted) |
| Intraclass coefficient | (MSB – MSE) /  (MSB + MSE) | MSB = mean square between; MSE = mean square error; MSE = SD d_scaled_ for duplicate measures |

**E. Reference Change Value (RCV)**

| **Scale** | **Formula** | **Comment** |
| --- | --- | --- |
| RCV | Z * √2 * CV (scaled) | CV is total of analytic and within-subject variation |
|  |  | Bias usually negligible if instrument is well calibrated |
|  |  | Estimates often conservative (e.g., Z = 1.96) |

**e-Table 4** Characteristics of the PAH Group

| Characteristic | PAH  (n = 221) |
| --- | --- |
| PAH Etiology |  |
| Idiopathic | 105 (48) |
| Connective tissue disease | 55 (25) |
| Porto-pulmonary | 14 (6) |
| Congenital disease | 4 (2) |
| Heritable (confirmed genetics) | 1 (0.5) |
| PAH mixed associated with left heart disease | 17 (8) |
| PAH mixed associated with chronic lung disease | 17 (8) |
| Mixed etiology | 8 (4) |
| Walk distance, m | 332 (172) |
| Treatment-naïve | 77 (35) |
| Mean arterial blood pressure, mmHg | 85 (13) |
| Mean pulmonary artery pressure, mmHg | 52 (14) |
| PAWP, mmHg | 11 (5) |
| Cardiac output, L/min | 3.8 (1.4) |
| Cardiac index, L/min/m^2^ | 2.0 (0.6) |
| PVR index, mmHg.min.m^2^/L | 23 (11) |
| NT-proBNP, pg/mL | 991(256-2497)* |
| eGFR, mL/min/1.73m^2^ | 55 (22) |
| Total bilirubin, mg/dL | 0.97 (1.01) |
| Hemoglobin, g/dL | 13.3 (2.3) |
| REVEAL Lite 2 risk score | 7.9 (3.1) |
| Death or transplant within 5 years | 78 (35) |

Data are presented as mean (standard deviation) or as count (percentage); *these data are presented as median and interquartile range. eGFR = estimated glomerular filtration rate; NT-proBNP = N-terminal pro-B-type natriuretic peptide; PAH = pulmonary arterial hypertension; PAWP = pulmonary arterial wedge pressure; PVR = pulmonary vascular resistance; REVEAL = Registry to Evaluate Early and Long-Term PAH Disease Management.

**e-TABLE 4b** Detailed Medication Profile

| Medication Class | | Overall N = 223 |  | | Therapy Complexity | | |
| --- | --- | --- | --- | --- | --- | --- | --- |
|  |  |  | **Monotherapy n = 87** | **Bi-therapy n = 38** | | **Tri-therapy n = 14** | **No Treatment n = 77** |
| No treatment before assessment | | 77 (35%) | 0 (0%) | 0 (0%) | | 0 (0%) | 77 (100%) |
| Prostacyclin analogs | | 65 (29%) | 17 (20%) | 34 (89%) | | 14 (100%) | 0 (0%) |
| Endothelin receptor antagonists | | 53 (24%) | 24 (28%) | 15 (39%) | | 14 (100%) | 0 (0%) |
| PDE5 inhibitors | | 77 (35%) | 38 (44%) | 25 (66%) | | 14 (100%) | 0 (0%) |
| sGC stimulators | | 2 (0.9%) | 0 (0%) | 2 (5.3%) | | 0 (0%) | 0 (0%) |
| Etiological/Other treatment* | | 8 (3.6%) | 8 (9.2%) | 0 (0%) | | 0 (0%) | 0 (0%) |
|  |  | | | | | | |

*Other treatment included calcium channel blockers. PDE5 = phosphodiesterase type 5; sGC = soluble guanylate cyclase.

**e-Table 5** Granular Quality Assessment of Peak TRV and Right Heart Parameters

|  |  |
| --- | --- |
| Ventricle Image Quality Grade | **Sample Size (n = 434)** |
| Good quality | 290 (67) |
| Moderate quality | 109 (25) |
| Low quality | 17 (4) |
| No consensus | 18 (4) |
| Atrium Image Quality Grade |  |
| Good quality | 322 (74) |
| Moderate quality | 80 (18) |
| Low quality | 3 (0.7) |
| No consensus | 29 (7) |
| TR Signal Image Quality Grade |  |
| Good to excellent quality | 192 (44) |
| Moderate quality | 118 (27) |
| Low quality (no signal) | 84 (19) |
| Indeterminate/no consensus | 40 (9) |

Data are n (%).
TR = tricuspid regurgitation.

**e-TABLE 6** Spearman Correlation of Deep Learning and Core Laboratory Reads

**a. Spearman Correlation Coefficients Across the Different Reads**

|  | **DL vs. CL1 (view-agnostic)** | **DL vs. CL2 (same- view)** | **CL2 vs. CL1** |
| --- | --- | --- | --- |
| Peak TRV | 0.90 | 0.94 | 0.93 |
| RV basal diameter | 0.76 | 0.87 | 0.88 |
| TAPSE | 0.78 | 0.83 | 0.94 |
| RA area | 0.86 | 0.96 | 0.89 |
| RVEDA | 0.86 | 0.94 | 0.93 |
| RVESA | 0.89 | 0.90 | 0.95 |
| RVFAC | 0.77 | 0.82 | 0.90 |

**b. Spearman Correlation PAH Cohort (DL vs. CL1 [View-Agnostic] Reader)**

| **Comorbidity** | **n** | **Peak TRV**  **(Spearman rho DL-CL)** | **p** | **RV basal diameter**  **(Spearman rho DL-CL)** | **p** |
| --- | --- | --- | --- | --- | --- |
| Entire group | 223(100%) | 0.78 | <0.001 | 0.74 | <0.001 |
| CREST syndrome | 22 (10%) | 0.58 | 0.008 | 0.65 | 0.003 |
| Liver disease | 23 (11%) | 0.82 | <0.001 | 0.82 | <0.001 |
| Mixed connective tissue disease | 17 (8.2%) | 0.58 | 0.02 | 0.88 | <0.001 |
| Obstructive lung disease | 28 (13%) | 0.93 | <0.001 | 0.67 | <0.001 |
| Restrictive lung disease | 25 (12%) | 0.90 | <0.001 | 0.83 | <0.001 |
| Rheumatoid arthritis | 11 (5.2%) | 0.72 | 0.03 | 0.64 | 0.12 |
| Scleroderma | 37 (17%) | 0.84 | <0.001 | 0.75 | <0.001 |
| Systemic lupus | 15 (7.1%) | 0.79 | <0.001 | 0.83 | 0.001 |

Model developed without an intercept where β represents the coefficient; CL = core laboratory; CL1 = view-agnostic core laboratory; CL2 = same-view core laboratory; DL = deep learning; RA = right atrial; RV = right ventricle; RVEDA = right ventricular end-diastolic areas; RVESA = right ventricular end-systolic area; RVFAC = right ventricular fractional area change; TAPSE = tricuspid annular plane systolic excursion; TRV = tricuspid regurgitation velocity.

**e-Table 7** Systematic Differences and Scaled Percentile Precision of the DL Versus CL2 Reader

| **Measure** | **Difference** | **Systematic Difference** | **Percentile Precision** |
| --- | --- | --- | --- |
| Peak TRV | Nominal, m/s | 0.18 (0.15-0.20) | 0.14 (0.13-0.16) |
|  | Relative, % | 6.73 (5.82-7.65) | 5.45 (4.68-6.0) |
|  | | | |
| RV basal diameter | Nominal, mm | -1.96 (-2.52 to -1.58) | 3.47 (2.94-3.78) |
|  | Relative, % | -5.24 (-6.37 to 3.90) | 7.24 (6.59-8.15) |
|  | | | |
| TAPSE | Nominal, mm | 0.45 (0.14-0.83) | 1.82 (1.63-2.08) |
|  | Relative, % | 2.00 (0.80-4.34) | 9.06 (8.03-10.83) |
|  | | | |
| RA area | Nominal, cm^2^ | -1.30 (-1.50 to -1.20) | 1.03 (0.91-1.17) |
|  | Relative, % | -8.27 (-9.01 to -7.41) | 4.77 (4.10-5.47) |
|  | | | |
| RVEDA | Nominal, cm^2^ | -0.70 (-1.00 to -0.30) | 1.84 (1.62-2.06) |
|  | Relative, % | -3.21 (-4.45 to -1.53) | 7.07 (6.04-8.24) |
|  | | | |
| RVESA | Nominal, cm^2^ | -0.70 (-0.90 to -0.40) | 1.78 (1.59-2.02) |
|  | Relative, % | -4.17 (-6.02 to -2.87) | 10.14 (8.84-11.57) |
|  | | | |
| RVFAC | Nominal, % | 2.44 (1.45-3.12) | 4.41 (3.77-5.06) |
|  | Relative, % | 6.97 (5.01-9.41) | 15.86 (13.43-18.34) |

Data are presented as median and 95% confidence interval. CL2 = same-view core laboratory; DL = deep learning; RA = right atrial; RV = right ventricular; RVEDA = right ventricular end-diastolic area; RVESA = right ventricular end-systolic area; RVFAC = right ventricular fractional area change; TAPSE = tricuspid annular plane systolic excursion; TRV = tricuspid regurgitation velocity.

**e-TABLE 8** Median and Percentile Range for Core Laboratory and Deep Learning Reads of the Healthy Cohort Compared to the World Alliance Societies of Echocardiography and American Society of Echocardiography Reference Limits and Thresholds

| **Variable** | **Sex** | **WASE/ASE^2-3^** | **CL1** | **CL2** | **DL (Us2.ai)** |
| --- | --- | --- | --- | --- | --- |
| Peak TRV, m/s | both | 2.1 (1.3-2.7)  (ASE: < 2.8) | 2.1 (1.7-2.5) | 2.0 (1.6-2.4) | 2.2 (1.8-2.7) |
| RV basal diameter index, mm/m^2^ | male  female | 18.4 (13.5-24.4)  18.7 (16.5-20.1)  (ASE: < 24) | 20.0 (15.6-26.4)  20.5 (15.7-26.3) | 20.1 (14.2-25.1)  20.6 (16.0- 25.8) | 20.3 (13.5-26.5)  20.6 (15.8-26.2) |
| RA area index, cm^2^/m^2^ | male  female | - | 8.7 (5.5-12.3)  8.5 (5.9-12.8) | 8.6 (6.1-12.7)  8.2 (5.8-11.8) | 8.5 (5.8-13.1)  8.1 (5.7-11.5) |
| RA volume index,  mL/ m^2^ | male  female | 20 (11-35)  18 (10-31)  (ASE < 43) | 24.0 (11.6-42.9)  20.3 (12.1-34.2) | 23.7 (13.0-44.0)  20.2 (12.1-33.9) | 22.9 (12.2-45.8)  19.9 (11.9-33.7) |
| TAPSE, mm | male  female | 21.9 (15.4-30.4)  21.6 (15.1-29.6)  (ASE > 17) | 24.5(20.0-32.0)  25.0 (19.9-30.8) | 24.0 (19.5-32.0)  24.0 (19.0-30.0) | 23.7 (16.6-31.1)  23.7 (16.0-30.5) |
| RVEDA index, cm^2^/m^2^ | male  female | 9.9 (6.1-13.7)  8.9 (5.5-12.3)  (ASE < 14) | 12.6 (7.9-16.5)  11.5 (7.6-15.6) | 11.6 (6.9-14.4)  10.5 (7.7-13.3) | 12.0 (7.0-16.5)  10.7 (6.8-14.2) |
| RVESA index, cm^2^/m^2^ | male  female | 5.7 (3.5-7.9)  5.1 (3.1-7.1)  (ASE < 8) | 7.8 (4.8-10.2)  6.9 (4.5-9.5) | 6.5 (3.9-9.3)  5.8 (4.0-7.9) | 7.0 (3.9-10.2)  5.9 (3.9-8.6) |
| RVFAC, % | male  female | 42.2 (35.2-50.2)  43.3 (35.3-51.1)  (ASE > 35%) | 39.0 (35.0-46.0)  40.0 (36.0-46.0) | 42.2 (35.3-49.7)  43.6 (37.0-51.0) | 43.6 (29.3-55.7)  46.0 (18.2-57.8) |

Data are presented as median and 95% confidence interval. ASE = American Society of Echocardiography; CL1 = view-agnostic core laboratory; CL2 = same-view core laboratory; DL = deep learning; RA = right atrial; REVESA = right ventricular end-systolic area; RV = right ventricular; RVEDA = right ventricular end-diastolic area; RVFAC = right ventricular fractional area change; TAPSE = tricuspid annular plane systolic excursion; TRV = tricuspid regurgitation velocity; WASE = World Alliance Societies of Echocardiography. Data presented as medians with it’s 5th-95^th^ confidence intervals.

**e-TABLE 9** Relative Precision Across the Range of Measures (DL vs. CL1 Reader)

| **Measure** | **Percentile Precision (%) Analyte Relationship** | **Example with Less Severity** | **Example with Worst Severity** | **Entire group** |
| --- | --- | --- | --- | --- |
| Peak TRV | 8.1 - 0.41 *TRV | 7.3% at 2 m/s | 6.5% at 4.0 m/s | 6.7% |
| RV basal diameter | 7.6 + 0.023 * RV basal diameter | 8.3% at 30 mm | 8.8% at 50 mm | 10.1%* |
| TAPSE | 16.2 - 0.28 *TAPSE | 9.5% at 24 mm | 12% at 15 mm | 11.5% |
| RA area | 8.4 + 0.05 * RA area | 9.2% at 16 cm^2^ | 9.7% at 26 cm^2^ | 10.2% * |
| RVEDA | 7.8 + 0.047 * RVEDA | 8.7% at 20 mm^2^ | 9.7% at 40 mm^2^ | 9.2% |
| RVESA | 16.3 - 0.21 *RVESA | 13.4% at 12 cm^2^ | 11.3% at 30 cm^2^ | 12.4% |
| RVFAC | 29.6 - 0.40 * RVFAC | 13.6% at 40% | 19.6% at 25% | 17.3% |

Model assumes linear quantile relationship. *When the quantile linear base method reports lower (better) precision than the entire group metric, this suggests non-linearity of relative precision at lower or higher measure value. CL1 = view-agnostic core laboratory; DL = deep learning; RA = right atrial; RV = right ventricular; RVEDA = right ventricular end-diastolic area; RVESA = right ventricular end-systolic area; RVFAC = right ventricular fractional area change; TAPSE = tricuspid annular plane systolic excursion; TRV = tricuspid regurgitation velocity.

**e-Table 10** AUC for the Discrimination of PAH Status with Fully Automated Echocardiography

| Echocardiographic Parameter | CL1 AUC | DL AUC | n (Common Reads) |
| --- | --- | --- | --- |
| Peak TRV, m/s | 0.99 (0.98-1.00) | 0.98 (0.97-1.00) | 370 |
| RV basal diameter index, mm/m | 0.98 (0.98-1.00) | 0.80 (0.75-0.85) | 341 |
| TAPSE, cm | 0.94 (0.91-0.97) | 0.79 (0.74-0.84) | 302 |
| RA area index, cm^2^/m^2^ | 0.76 (0.71-0.81) | 0.74 (0.68-0.79) | 370 |
| RVEDA index, cm^2^/m^2^ | 0.90 (0.87-0.93) | 0.85 (0.81-0.89) | 331 |
| RVESA index, cm^2^/m^2^ | 0.96 (0.94-0.98) | 0.92 (0.89-0.95) | 331 |
| RVFAC, % | 0.99 (0.99-1.00) | 0.90 (0.86-0.93) | 313 |

Data are presented with 95% confidence intervals. All of the measures significantly differentiated the control group from PAH. AUC = area under the curve; CL1 = view-agnostic core laboratory; DL = deep learning; PAH = pulmonary arterial hypertension; RA = right atrial; RV = right ventricular; RVEDA = right ventricular end-diastolic area; RVESA = right ventricular end-systolic area; RVFAC = right ventricular fractional area change; TAPSE = tricuspid annular plane systolic excursion; TRV = tricuspid regurgitation velocity.

**e-FIGURE 2** Estimated Right Heart Catheterization Versus Invasively Measured Pulmonary Systolic Pressure in DL Versus Controls

**
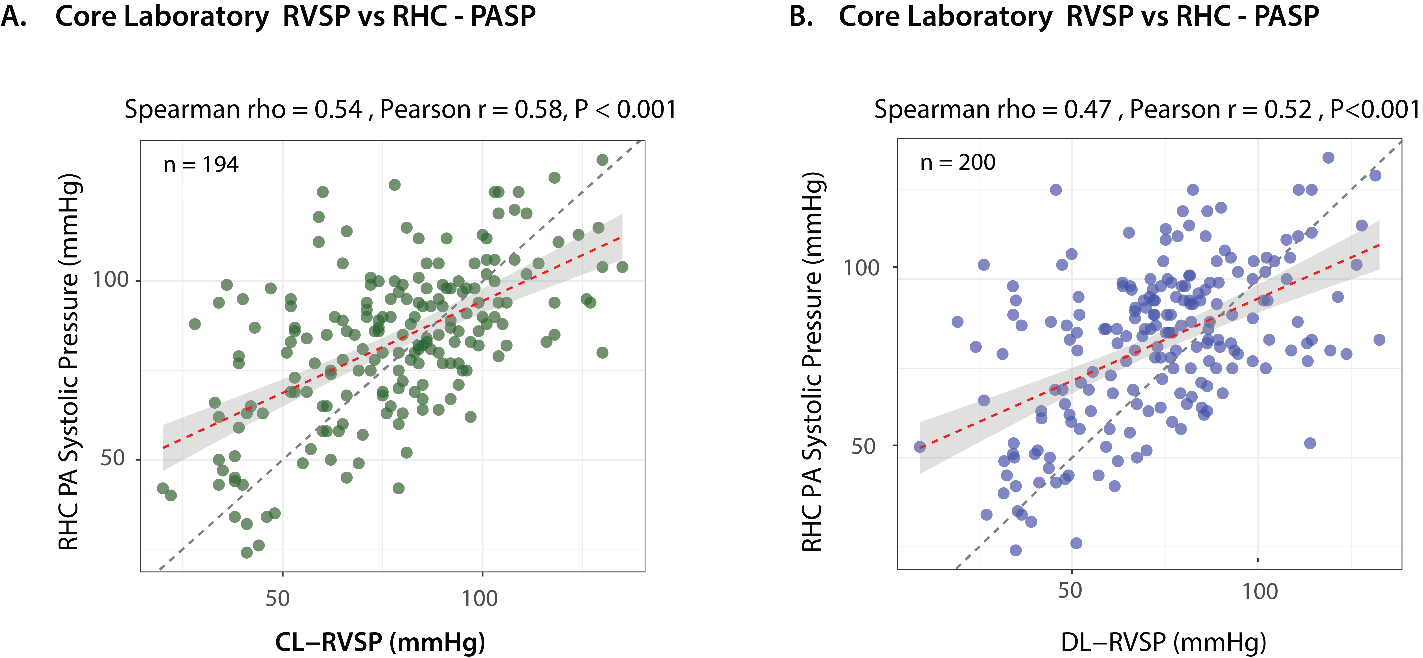
**

CL = core laboratory; DL = deep learning;; PASP = pulmonary artery systolic pressure; RHC = right heart catheterization; RVSP = right ventricular systolic pressure.

**e-TABLE 11** Demographic, Hemodynamic, and Baseline Echocardiographic Features or Clinical Laboratory Reads

| **Characteristic** | **Referral Cohort (Mean TRV) (n = 196)**  **Mean (SD)** | **With Peak TRV in Both Cohorts (n = 171)**  **Mean (SD)** |
| --- | --- | --- |
| Age (years) | 62 (16) | 63.2 (16) |
| Sex (male), n (%) | 93 (47) | 76 (44) |
| Race, n (%) | White: 113 (58)  Others or unknown: 45 (23)  Asian: 21 (11)  Native Hawaiian: 9 (5)  Black: 8 (4) | White: 99 (58)  Other or unknown: 41 (24)  Asian: 22 (13)  Native Hawaiian: 6 (3)  Black: 3 (1) |
| Right heart catheterization |  |  |
| No PH, n (%) | 89 (45) | 75 (44) |
| Mild PH, n (%) | 107 (55)  Pre-capillary PH: 37 (34)  Ipc PH: 21 (20)  Cpc PH: 49 (46) | 96 (56)  Pre-capillary: 37 (39)  Ipc PH: 18 (19)  Cpc PH: 41 (43) |
| MPAP, mmHg | 21.0 (7.1) | 21.3 (7.0) |
| PAWP, mmHg | 11.2 (6.0) | 11.1 (6.0) |
| PVR, Wood units | 2.1 (1.7) | 2.2 (1.7) |
| Echocardiography clinical laboratory read |  |  |
| LVEF, % | 62.1 (6.5) | 61.7 (6.6) |
| LVEDV, mL | 86.0 (37.0) | 84.4 (36.8) |
| LVESV, mL | 32.3 (14.5) | 32.0 (14.3) |
| LVIDd, cm | 4.6 (0.6) | 4.6 (0.6) |
| Peak TRV, m/s | 2.7 (0.5) | 2.7 (0.5) |
| Echocardiography DL read |  |  |
| LVEF, % | 61. 1 (7.6) | 61.0 (7.6) |
| LVEDV, mL | 92.7 (34.0) | 91.4 (32.7) |
| LVESV, mL | 36.2 (16.2) | 35.7 (16.0) |
| LVIDd, cm | 4.4 (0.6) | 4.4 (0.6) |
| Peak TRV, m/s | 2.8 (0.6) | 2.8 (0.6) |
| RVIDd, cm | 3.8 (0.7) | 3.8 (0.7) |
| RA area, cm^2^ | 16.3 (6.2) | 16.4 (6.2) |
| RVFAC, % | 42.0 (12.0) | 42.4 (11.5) |

Cpc PH = combined pre- and post-capillary pulmonary hypertension; DL = deep learning; Ipc PH = isolated post capillary pulmonary hypertension; LVEF = left ventricular ejection fraction; LVEDV = left ventricular end-diastolic volume; LVESV = left ventricular end systolic volume; LVIDd = left ventricular internal diameter during diastole; MPAP = mean pulmonary artery pressure; PAWP = pulmonary artery wedge pressure; PH = Pulmonary hypertension; PVR = pulmonary vascular resistance; RA = right atrial; RV = right ventricular; RVFAC = right ventricular fractional area change; RVIDd = right ventricular internal diameter during diastole; TRV = tricuspid regurgitation velocity

**e-TABLE 12** Supporting Criteria for the Diagnosis of Pulmonary Hypertension

| **Supporting Criteria (Clinical Read)** | **Supporting Criteria (DL)** |
| --- | --- |
| RA size enlarged | RVIDd/LVIDd > 1 |
| RV size enlarged | TAPSE/RVSP < 0.55 mm/mmHg (reduced) |
| Septal flattening | RA area (s) > 18 cm^2^ (enlarged) |
| RV function reduced | PA AT < 105 m/s (shorter) |
| PA size enlarged | e-RAP > 3 mmHg |
| e-RAP > 3 |  |

List of the supporting criteria added to the logistic regression models for clinical reads and deep learning reads. As per European Society of Cardiology/European Respiratory Society guidelines, having at least two of any of these criteria fulfills the presence of supporting criteria.
e-RAP = estimated right atrial pressure; LVIDd = left ventricular internal diameter during diastole; PA = pulmonary artery; PA AT = pulmonary artery acceleration time; RA = right atrial; RV = right ventricle; RVIDd = right ventricular internal diameter during diastole; RVSP = right ventricular systolic pressure; TAPSE = tricuspid annular plane systolic excursion.
